# The Black Pedagogy Scale: A New Task to Explore Educational Practices for Children’s Well-Being

**DOI:** 10.5964/ejop.v16i2.1876

**Published:** 2020-05-29

**Authors:** Eleonora Florio, Letizia Caso, Ilaria Castelli

**Affiliations:** a Department of Human and Social Sciences, University of Bergamo, Bergamo, Italy; The Maria Grzegorzewska University, Warsaw, Poland

**Keywords:** Black Pedagogy, Poisonous Pedagogy, psychological maltreatment, disciplinary practices, authoritarian, child-rearing methods

## Abstract

The present contribute focuses on the concept of “Black Pedagogy” (Rutschky, 1977; ISBN: 3548356702), meant as a set of educational practices assimilable into those that nowadays are included in the frame of physical and psychological maltreatment (e.g., corporal punishment, frightening children, etc.). The purpose of this work is to present our operationalization proposal of the concept and the results deriving from a first validation of the “Black Pedagogy Scale”. The questionnaire was administered to 374 Italian university students in their university classrooms (pilot study with double administration) and to 830 Italian adults, parents of primary school-aged children, through an online survey platform (main study). In the pilot study, explorative analyses, paired-samples t-test and ML EFA (with Varimax rotation) were performed. In the main study, proprieties of the refined instrument and relations between the construct of Black Pedagogy and demographics were explored. The Black Pedagogy Scale (α > .8) resulted composed by three factors, consistently with what was initially hypothesized: “Values of Black Pedagogy” (var. 18.7%), “Education of children over time” (var. 10.6%) “Methods of Black Pedagogy” (var. 8.6%). Participants resulted more in agreement with Black Pedagogy’s values rather than with its methods, and those with higher educational qualification showed less agreement with the construct, F(2, 813) = 28.22, p < .001, η² = .065. The possible legacy of a Black Pedagogy’s forma mentis can contribute to explain why some detrimental disciplinary practices are culturally deemed as acceptable. Results suggest designing interventions focused on educational values to discourage such practices.

## Origin of the Concept

The concept of *Schwarze Pädagogik*, literally meaning “Black Pedagogy” slowly spread in discourses about child-rearing and child-education, and laid the foundations for interdisciplinary reflections connecting pedagogy and social-juridical psychology fields of study. The first appearance of the concept can be traced back to the work of Katharina Rutschky (1977), who gathered various sources from the eighteenth and nineteenth century with the precise purpose to show which were the considerations, values and practices promoted by pedagogists and physicians of those times and to problematize the “scientified” and socialized education through a historical, and critical, reflection on education as part of the civilization process (Rutschky, 2015). The author argues indeed that “education” is a bourgeois phenomenon belonging to modernity and, instead of focusing on progresses and innovations arising from the Enlightenment, she puts into the foreground the actual repercussions of educational principles and socio-cultural context of that time on the daily life of children, corroborated by the multitude of primary sources that the author collects in her *florilegium* of Black Pedagogy. The result is the description of a systematic use of power, violence and intimidation to “train” children in bourgeois virtues such as diligence and subordination (Brokate, 2005). Polarization of power in favor of adults is in fact the bedrock of Black Pedagogy, something that is immediately reflected on the resulting educational practices consisting in physical and psychological violence, control, surveillance, oppression and punishment (Kühn, 2014; Rutschky, 2015). Miller (1980) provided a more systematic definition of the concept combining it with a psychological explanation of its foundations in the mind of educators and of its consequences on children, and vice versa: in fact, according to the author, such child-rearing culture creates a vicious circle of subtle and explicit violence that is transmitted through generations. In the English version of her work, Miller (1983) refers to Black Pedagogy as to “poisonous pedagogy”, nonetheless in the present work it has been decided to use the label “Black Pedagogy” in order to maintain a direct semantic connection with the original term, which was also used as such by Alice Miller in her original publication (Miller, 1980), and it has been translated into Italian language maintaining the reference to the black color (Miller, 2007; Rutschky, 2015). It is necessary to strongly underline that this term is in no way referring or connected to “Black Pedagogy” meant as the education provided to black students or the implementation of black studies in schools’ curricula (Johnson, Pitre, & Johnson, 2014; Pitre, Ray, & Pitre, 2008): the semantic adherence to the originally coined term has been considered a priority, trusting that the clear disambiguation provided is sufficient to distinguish the different conceptual areas. Therefore, in the context of the present work, the label “Black Pedagogy” represents the systematic use of educational methods focused on the primary objective of breaking the child’s will and to shape the child’s character according to the ideal values of educators and society: some of its more recognizable characteristics are discipline, the safeguard of educator’s authority, strict rules, as well as control and power of the educator over the child (Kühn, 2014). Some examples of Black Pedagogy’s methods are beating the child, using subterfuges and manipulation as parenting techniques, and humiliating or ridiculing the child (Miller, 1983).

## Current Functionality of the Construct

An idea of the child as subject of rights and the cultural valorization of childhood emerges relatively recently, starting from the 1960s (Di Blasio, 2000) and culminating in the 1989 with the *Convention on the Rights of the Child*, ratified in Italy in 1991 with Law 176/91. The Convention states the need to protect the child from “all forms of physical or mental violence” (UN General Assembly, 1989, article 19, paragraph 2) providing a broad and all-embracing definition of violence against children. Nonetheless, different countries vary in the reported normativeness of physical discipline (Lansford et al., 2005) and, for this reason, there could have been a certain difficulty for individuals (teachers, parents, etc.) in recognizing and defining the very limit between a correct disciplinary practice and a detrimental one, due to specific cultural assumptions. In fact, the Committee on the Rights of the Child issued a general comment on the aforementioned article 19 “since the extent and intensity of violence exerted on children is alarming” and reminded that “no violence against children is justifiable; all violence against children is preventable” (UN Committee on the Rights of the Child, 2011, p. 3). Therefore, the Committee provided a more detailed definition of the different forms of violence against children, among which mental and corporal violence result particularly similar to Black Pedagogy methods. In fact, mental violence (i.e., psychological maltreatment or emotional abuse) is described as all forms of iterated harmful interactions that convey to the child a sense of worthless, that are focused on scaring, threatening, humiliating and isolating, as well as denying emotional responsiveness, exploiting, rejecting and ignoring. Corporal punishment is instead referred to “any punishment in which physical force is used and intended to cause some degree of pain or discomfort, however light. It mostly involves hitting (‘smacking’, ‘slapping’, ‘spanking’) children, with the hand or with an implement” (UN Committee on the Rights of the Child, 2011, paragraph 24). Physical violence for disciplinary purposes (e.g., harsh treatment, and cruel or humiliating punishment) is still common in the context of families both in industrialized and in developing countries (Durrant, 2005; Pinheiro, 2006), and it often coexists with psychological forms of violence harmful to children’s well-being both in domestic and in school contexts (Pinheiro, 2006). According to Perticari (2016), who edited the Italian translation of Katharina Rutschky’s *Schwarze Pädagogik*, this level of children abuse is a contemporary problem and it deserves urgent attention, since this pathogenic education built on a devious authoritarian mentality is infecting children’s everyday life and disguising abuse as a form of love and care. The author specifies that such level of maltreatment is covert, difficult to recognize and also very complicated: most adults do not realize they behave in a harmful way when slapping, yelling at kids or humiliating them, and they are instead convinced that they are acting in children’s best interests. In fact, such disciplinary measures are considered necessary to promote a healthy and robust upbringing and, consequently, adults tend to justify or minimize them, often not recognizing them as something from which children have to be protected.

In the face of such considerations, a reference to the Italian legislation on the subject is necessary: before the reform of Family Law occurred in Italy in 1975, the abrogated article 319 of Italian Civil Code (I.C.C.) explicitly acknowledged the parent’s power of restraining a child’s misconduct, and it was interpreted as a sort of exemption from responsibility for the harmful acts committed by the parent towards the child if they were aimed at repressing bad behaviors, this being an area of exemption connected to the *ius corrigendi* (i.e., the right to correct), a corollary of parental *potestas* (Paladini, 2012). The existence of *ius corrigendi* is deduced from Art. 571 of Italian Penal Code (I.P.C.) that defines the offense of “Abuse of means of correction or discipline”. The fact of referring to an abuse implies that there is a legitimate and permitted use of disciplinary measures, which can result in abuse if the measure is excessive, arbitrary or untimely (Ferraro, 2008). Moreover, the literature reports that with specific reference to Italian family relationships, part of the doctrine considers *vis modica* (i.e., moderate violence) a licit means of correction and also that it would be difficult to imagine the prospect of completely banishing it from family context (Catullo, 2012; Tortorelli, 2014). It seems therefore important to ponder further on two aspects: firstly, exploring whether and how the distinction between licit and illicit means of correction may be clear in the minds of educators – both parents and teachers –. Secondly, verifying if and how they can realize they are mistaken when applying such educational practices (e.g., a slap or a verbal insult) that they have seen widely adopted by the previous generations (parents, grandparents, etc.) without questioning their legitimacy. These two aspects are based on the hypothesis that the ongoing practice of the subtlest forms of disciplinary physical or mental violence coincides with a persistence in our society of the hierarchical and authoritarian model of the family mentioned above, which appears to be well described by Black Pedagogy values. Therefore, a measuring instrument capable of grasping such authoritarian educational model was needed. At first, we considered using the already existing “Poisonous Pedagogy Scale” within the O’Brien Multiphasic Narcissism Inventory (O’Brien, 1987), a subscale elaborated on the basis of Alice Miller’s definition of Black Pedagogy. It measures the belief of having the ability to control others by taking advantage of one’s own superordinate position (Montebarocci et al., 2003; Sines, Waller, Meyer, & Wigley, 2008). Nevertheless, we decided not to use such a scale and to develop a new one, in order to include more detailed and varied aspects of the Black Pedagogy concept, in line with the research interests of the present study.

## Purposes of the Study

This work is part of Doctoral thesis (Florio, 2018): the main aim of this part of the research is to elaborate the Black Pedagogy Scale, a new instrument that carefully gathers the values and methods of Black Pedagogy, in order to explore the possible presence of this unexpressed legacy in our territory. In our view, this is important for children’s well-being, because the presence of such methods in educational contexts could hamper the complete abandon of physically and psychologically harmful disciplinary practices and undermine the course of establishing of a more positive adult-child relationship. Secondly, we present the results of a first validation study of our Black Pedagogy Scale, i.e. a pilot study carried out on university students to refine the Black Pedagogy Scale, and the results of a new research on caregivers –parents of Primary school-age children– in order to explore the presence of Black Pedagogy in the context of our territory.

## Method

### Instrument

From the scientific literature on Black Pedagogy, we individuated 41 fundamental statements describing Black Pedagogy’s values, practices and main convictions, and we elaborated them according to the general rules of items’ construction (Chiorri, 2011). Two main sections composed the instrument in its first version: “Black Pedagogy Observation” (BPO hereinafter) and “Estimations of Black Pedagogy Diffusion” (ED). The BPO explores the construct of Black Pedagogy as it has been described in the literature, with 41 items (e.g., “Bad habits and character flaws must be eliminated through education”, “Children should be kept constantly under control”, and “Children must learn to be humble”) and a response set designed as an agreement 4-point Likert scale. Therefore, participants were asked to take a clear stance for or against each statement, and a possible change in the response scale would have been evaluated after analyzing missing data patterns. The ED section instead, gathers the estimations of the current diffusion of Black Pedagogy practices on our territory, and of the diffusion of these practices in the past. Therefore, a forty-second item (i.e., “By educational means ‘used in the past’ are meant the educational practices that took place in Italy from the post-World War II period until the 1980s”) was added, in order to assess the time period to which participants referred when thinking of “the past”, namely when such practices were widespread. This second section consists of two identical 12-items lists of the disciplinary practices typical of Black Pedagogy educational style (e.g., “Pedagogical beating (slaps, caning, etc.)” and “Treating the child coldly as a consequence of his/her disobedience”): participants have to evaluate the diffusion of each method one first time in respect to the past, and a second time in respect to the present days. Items of the second section were also accompanied by a 4-point Likert scale response set, but based on frequency instead of agreement. Clear instructions were given in order to inform respondents of the change in response options meanings (1 = “Not present at all”; 2 = “Present, but not common”; 3 = “Present”; 4 = “Widespread”).

### Participants and Procedures

Participants of the pilot study were Italian university students of the Department of Human and Social Sciences in Northern Italy. Most of them were attending the first year of their degree course in Sciences of Education (93.9% at Time 1; 94.4% at Time 2). We chose to include only students of the first year so that they could be still informative about their own folk beliefs concerning educational practices as they had developed them throughout their own life experience, and not as they have been taught about in University courses of Psychology and Pedagogy. The Black Pedagogy Scale was administered in two different occasions to the same group of subjects in order to assess if there were changes in the answers over time. We expected a stability of answers across time since Black Pedagogy represents a body of values and beliefs that should not change unless training or intervention occurs. The two administration sessions took place with a temporal distance of two weeks and have been organized directly in students’ university classrooms, as well as the restitution of results, which has been designed as an interactive reflection with participants. Table 1 summarizes sample descriptives of both administration sessions: as can be noticed, there is a considerable decrease in the number of participants from Time 1 to Time 2. This is possibly due to the normal decrease of class attendance across the semester.

**Table 1:** Sample Descriptives of Pilot Study

Variable	Time 1	Time 2
*N*	374	251
Males	7.8%	11.6%
Females	92.2%	88.4%
Age		
Min	18	18
Max	42	35
*M*	20	20
*SD*	2.6	2.6
Secondary Education Diploma^a^	97.3%	96.0%
Participants working	45.5%	43.4%
Participants working with children	25.7%	25.9%
Participants in contact with children in personal life	98.1%	98.4%

^a^Lowest educational qualification.

In the main study, the final version of Black Pedagogy Scale has been administered through an online survey platform to a sample of 830 adult subjects, recruited in the context of Northern Italy primary schools with the support and consent of school Head Teachers. The following table (Table 2) presents the sample descriptives of the main study.

**Table 2:** Sample Descriptives of Main Study (N = 830)

Variable	Percentage
Mothers	72.3%
Fathers	27.7%
Age		
Min	24
Max	64
*M*	41.5
*SD*	4.9
Parents of an only child	69.5%
Parents of two children	26.0%
Parents of three children	3.9%
Parents of four children	0.6%
Lower secondary school qualification	24.7%
Upper secondary school qualification	50.0%
University Diploma^a^	3.0%
Bachelor’s degree	10.7%
Master’s degree	6.0%
Post-Master’s specialization degree	3.9%
Participants involved in a sentimental relationship^b^	94.1%
Italian nationality	96.0%
Foreigners	3.1%
Double citizenship	0.6%

^a^Italian qualification established by Law 341/90, no longer in force. ^b^Stable relationship, cohabitation, married, or remarried. The remaining 5.2% was not involved in a sentimental relationship (i.e., single, separated, divorced or widow).

Both in the pilot and main study, participants were clearly informed about the objectives and phases of the research, about their rights as participants including the guarantee of anonymity and the possibility to drop-out of the study at any moment. All participants were treated in accordance with the Declaration of Helsinki (World Medical Association, 2008), as well as with the ethical guidelines for research provided by American Psychological Association (American Psychological Association, 2017) and by Italian Psychological Association (Associazione Italiana di Psicologia, 2015). Participants have had the possibility to receive further information following their questions and, afterwards, they were asked to express their informed consent in order to proceed to fill out the questionnaire.

## Results

### Results of the Pilot Study

For what concerns the pilot study, a first exploration of the proprieties of 41-items BPO section was conducted in order to investigate its reliability and the distribution of responses. Table 3 presents a summary of the exploration of BPO section (minimum score 41 - maximum score 164) at Time 1 and at Time 2. The mean was similar to the 5% Trimmed Mean, thus indicating that it was not necessary to exclude outliers. In terms of mean response, the result was a value of 2.5 both at Time 1 and at Time 2 (minimum response 1 - maximum response 4). The Little’s MCAR Test was not significant (*p* > .05) thus suggesting that data were missing completely at random. Cronbach’s α resulted in an adequate value of .83 at Time 1 and .82 at Time 2, suggesting reliability of the scale (DeVellis, 2016). The distribution is slightly heavy-tailed (Westfall, 2014) and characterized by a positive asymmetry towards lower values of the scale in both occasions.

**Table 3:** BPO Section: Results of Exploration Analyses at Time 1 and at Time 2

Analysis	Time 1	Time 2
*N*	338	232
*M*	104.14	102.72
*SD*	11.07	10.53
5%Trimmed *M*	103.96	102.44
Average response	2.54	2.51
Cronbach’s α	.83	.82
Skewness	.275	.348
Kurtosis	-.094	-.195
Kolmogorov-Smirnov normality test	*p* < .01	*p* < .05
Shapiro-Wilk normality test	*p* > .05	*p* < .05
Little’s MCAR test	*p* > .05	*p* > .05

At Time2, both Kolmogorov-Smirnov and Shapiro-Wilk normality test resulted significant (*p* < .05) thus suggesting violation of normality assumption. Nevertheless, values of skewness and kurtosis included between a range of -1 and +1 are considered acceptable (Muthén & Kaplan, 1985) and the histogram, as well as the Normal Q-Q Plot, showed that responses were reasonably normally distributed.

After Time 2, a paired samples *t*-test on 104 subjects was carried out for the comparison of the scores obtained on the BPO section at Time 1 (*M* = 103.13, *SD* = 11.07) and at Time 2 (*M* = 102.7, *SD* = 11.04). This resulted as not significant, *t*(103) = 0.713, *p* = .477 (two-tailed), with a strong positive correlation (*r* = .85, *p* < .001) between the results of the two administrations, thus suggesting that what the scale is measuring remains stable and consistent over time. The unremarkable decrease of the mean was 0.423 with a 95% confidence interval ranging from -0.753 to 1.6 and a Cohen’s *d* of 0.09.

The condition of our data, i.e. violating normality but reasonably normally distributed, is commonly encountered in social sciences, and Maximum Likelihood (ML) approach has been chosen since it is still recommended when a sever violation of normality is not present (Costello & Osborne, 2005; Fabrigar, MacCallum, Wegener, & Strahan, 1999). Data resulted adequate for factor analysis since Kaiser-Meyer-Olkin value was .79 (Kaiser, 1970, 1974) and Bartlett’s (1954) Test of Sphericity was significant (*p* < .001). Subsequently, ML Exploratory Factor Analysis (EFA) with Varimax rotation has been performed. After the first output of EFA it was decided to force the extraction of three factors following Kaiser’s criterion of eigenvalues > 1 (Kaiser, 1960) and on the basis of the inspection of the Scree Plot, which clearly showed a change of direction after the fourth dot (Cattell, 1966). Subsequently, cross-loadings items and items loading < .35 have been excluded, thus reaching a factorial structure (shown in Table 4) that explained a total variance of 37.9%.

**Table 4:** BPO Section: Final Results of Maximum Likelihood Exploratory Factor Analysis (With Varimax Rotation)

Item number	Factor 1	Factor 2	Factor 3
25	.590		
27	.551		
17	.539		
23	.539		
14	.533		
6	.517		
12	.465		
28	.442		
33	.419		
13	.411		
22	.395		
30	.392		
34		.762	
1		.665	
9		.627	
8 (reversed)		.571	
16 (reversed)		.433	
21			.591
36			.506
26			.485
38			.465
31 (reversed)			.447
41			.369
32			.353

*Note*. Extraction method: Maximum Likelihood; Rotation method: Varimax with Kaiser Normalization; Rotation converged in four iterations; Cross-loading items and items loading < .35 have been discarded.

According to the meaning of the 24 items included in the factorial structure, the three factors extracted have been entitled as follows: “Values of Black Pedagogy” (explained variance: 18.74%), collecting items concerning the main educational values and objectives typical of Black Pedagogy’s perspective; “Education of children over time” (explained variance: 10.62%), which refers to those items regarding attitudes towards the changes in children’s education (where adopting a Black Pedagogy’s perspective implies to be nostalgic about educational practices used in the past, because considered more effective and useful); “Methods of Black Pedagogy” (explained variance: 8.57%), which collects the items on Black Pedagogy's disciplinary and educational methods used as means to pursue its values and objectives.

Table 5 shows exploration analyses of the three different factors. It is interesting to notice that the mean response on “Methods of Black Pedagogy” is clearly lower than the one on “Values of Black Pedagogy”, as expected.

**Table 5:** Three Factors of BPO Section: Results of Separated Explorations (Pilot Study)

Factor	% of explained variance	Number of items	Cronbach’s α	*sk*	*ku*	K-S normality test	Mean response (min = 1; max = 4)
“Values of Black Pedagogy”	18.74	12	.79	.041	.119	*p* < .01	2.9
“Education of children over time”	10.62	5	.76	.103	.005	*p* < .001	2.8
“Methods of Black Pedagogy”	8.57	7	.67	.255	-.68	*p* < .001	1.9

An independent samples *t*-test was performed to compare scores on BPO section between those who work with children (*M* = 102.66, *SD* = 11.8) and those who do not (*M* = 100.4, *SD* = 10.62). The difference resulted not statistically significant, *t*(99) = 1, *p* = .32. Spearman’s rho coefficient between scores on BPO section and age showed a negative low correlation of -.142 (*p* < .05), thus suggesting a slight decrease in the agreement with Black Pedagogy construct with the increase of age. This result is certainly counter-intuitive if the reference literature is taken into account, but it has to be considered both in the light of the context of sample recruitment and of the fact that age distribution in the pilot study was very highly skewed towards lower values.

Item 42 is dedicated to temporal collocation and presented results that were quite challenging, since 61.1% of participants clearly agreed with this collocation, but the 38.9% was not completely satisfied with the temporal definition proposed by the item. This result suggested a reformulation of the item to help identifying what period of time participants have in mind when referring to the pedagogical practices used “in the past”.

The comparison between the diffusion of Black Pedagogy’s practices in the past and nowadays revealed that participants report a general decrease in the use of such educational measures except for what concerns the practices of blackmailing to control children actions (method “m” in Table 6) and of justifying unpleasant educational measures by telling the child that these are applied for his/her own good (method “n” in Table 6). Table 6 summarizes the mean response for each Black Pedagogy method and the significant level of average differences in the comparison between past and present diffusion.

**Table 6:** Mean Responses About the Diffusion of Black Pedagogy’s Practices in the Past and Nowadays

Black Pedagogy method	Past	Today	Mean difference	*p*
a. Pedagogical beating	3.48	2.19	1.29	< .001
b. Denial of a meal	3.04	1.34	1.70	< .001
c. Cautionary tales	2.97	2.38	0.59	< .001
d. Providing false information	3.09	2.69	0.4	< .001
e. Treating the child coldly	2.93	2.46	0.47	< .001
f. Toughening children up	2.8	1.35	1.45	< .001
g. Monitoring/discouraging sexuality	2.96	2.18	0.78	< .001
h. Lying by exacerbating consequences	2.94	2.57	0.37	< .001
i. Humiliating	2.74	1.9	0.84	< .001
l. Physical violence	3.05	1.67	1.38	< .001
m. Blackmailing	2.76	2.59	0.17	< .01
n. Unpleasant measures for children’s own good	2.92	2.97	-0.05	> .05

*Note*. 1 = “Not Present at all”; 2 = “Present, but not Common”; 3 = “Present”; 4 = “Widespread”.

Observing average comparisons, it emerges that mean differences are less pronounced for those educational practices that do not involve a physical type of harm to the child, culminating in the approximatively same level of current diffusion for what concerns method “m” and in a not significantly greater diffusion today than in the past of method “n”.

Finally, bivariate correlations have been performed between the three factors and the results on the lists of ED section. The only significant correlation was found between the scores on “Values of Black Pedagogy” and the evaluation of the diffusion of Black Pedagogy practices in the past (*r* = .18, *p* < .01): a low positive correlation suggesting that those who observed a higher diffusion of Black Pedagogy practices in his/her past experience tended also to score higher on the “Values of Black Pedagogy” factor. One possible interpretation of such result could be that those who have been in contact (even only as observers) with such practices tend to assimilate Black Pedagogy’s values and objectives, thus supporting the idea of an intergenerational transmission of physically and mentally violent disciplinary practices (Miller, 2007; Perticari, 2016). Since the sample of pilot study was homogeneous with regard to gender, educational qualifications and age, no specific analyses were conducted involving these variables.

### Results of the Main Study

Following the results of the pilot study, the Black Pedagogy Scale has been adjusted to elaborate a final version, which was administered to the participants of the main study (*N* = 830). Analyses on this second dataset replicate the exploration of scale proprieties but focus in particular on the investigation of the differences between the subgroups created on the basis of demographic information. To this purpose, *t*-tests, bivariate correlations and ANOVAs have been performed, according to the suitability of data for each specific analysis. The Black Pedagogy Scale in its final version (with a minimum possible score = 24 and a maximum possible score = 96 in BPO section) was administered to a sample of 830 adult subjects, parents of primary school-age children. The mean score was 60.33 (*SD* = 8.1, 95% CI [59.78, 60.88]), with a 5% Trimmed Mean of 60.36 and an average response of 2.5 (min = 1, max = 4). Both skewness and kurtosis were close to zero (*sk* = -.062, *SE* = .085, *ku* = .009, *SE* = .170) and Shapiro-Wilk normality test was not significant (*p* > .05). Cronbach’s α resulted .87, indicating reliability of the scale (DeVellis, 2016). Table 7 summarizes the results of separated exploration analyses of the three different factors: responses show that the agreement with “Methods of Black Pedagogy” is clearly lower than in the case of the other two factors, as emerged in the pilot study.

**Table 7:** Three Factors of BPO Section: Results of Separated Explorations (Main Study)

Analysis	“Values of Black Pedagogy” (Factor 1)	“Education of children over time” (Factor 2)	“Methods of Black Pedagogy” (Factor 3)
Number of items	12	5	7
Min	18	7	7
Max	47	20	26
*M*	34.51	13.58	12.24
*SEM*	.16	.09	.11
*SD*	4.64	2.51	3.06
95% CI			
*LL*	34.19	13.41	12.03
*UL*	34.82	13.76	12.44
5% Trimmed Mean	34.54	13.58	12.16
Average response (min = 1, max = 4)	2.89	2.72	1.75
Skewness	-.062	.056	.353
Kurtosis	.344	-.262	-.163
Cronbach’s α	.87	.75	.74
*p* (Shapiro-Wilk normality test)	< .001	< .001	< .001

*Note*. CI = confidence interval; *LL* = lower limit; *UL* = upper limit.

Pearson Product-moment correlation between BPO section and age resulted negative, weak, and significant (*r* = -.073, *p* < .05), thus suggesting again that there is slight decrease in scores on BPO section with the increase of age. Nonetheless, no significant correlations have been found between age and BPO subscales. The continuous scale of age has been collapsed into two age groups, since the cases of under 30 years and over 50 years of age were very few (4.1%), and an independent samples *t*-test was conducted to compare scores on BPO for the age group under 40 years and for the age group over 40 years. Unequal variances have been assumed since Levene’s Test for Equality of Variances was significant (*p* = .002), and a statistically significant difference has been found between the scores of participants aged under 40 years (*M* = 61.06, *SD* = 7.32) and those of participants aged over 40 years (*M* = 59.69, *SD* = 8.66): *t*(822.5) = 2.469, *p* = .014. Another independent-samples *t*-test was performed to compare BPO scores for parents of an only child and for parents of more than one child. A significant difference has been also found in the comparison of these two conditions, indicating that scores of parents of one child (*M* = 60.74, *SD* = 8.22) were significantly higher than those of parents with more children (*M* = 59.40, *SD* = 7.73): *t*(828) = 2.2, *p* = .028. A first attempt to interpret this unexpected result could be that parents of one child rely more on culturally learned methods of parenting practice, whereas the increased experience, effort, and/or relational complexity resulting from having two or more children lead parents to deviate from their educational legacy and to work on their own parenting solutions. This is a possible interpretation that surely needs to be further investigated in future studies. In fact, according to Edwards (2014) parenting experience (i.e., raising singletons or more than one child) is a variable that deserves more attention: in the context of her study, the author found that it is more likely for mothers raising two or more children to perceive their role in supporting their children’s emotional development and to be open to the idea of seeking professional behavior-related advice.

No significant difference was found instead between BPO scores of mothers (*M* = 60.12, *SD* = 8.21) and of fathers (*M* = 60.88, *SD* = 7.78): *t*(828) = -1.213, *p* = .225.

After assessing assumptions of normality and homogeneity of variance, a one-way between groups analysis of variance was conducted to explore the impact of educational qualification level on BPO scores. Participants have been divided in three groups according to their educational qualification (lower secondary education, upper secondary education and higher education). There was a statistically significant difference at the *p* < .001 level in BPO scores for the three groups: *F*(2, 813) = 28.22, *p* < .001. The effect size, calculated through Eta squared, resulted .065, representing a medium effect according to Cohen (1988). Post-hoc comparisons using the Tukey HSD test showed that the mean score for the group of participants with lower secondary education diploma (*M* = 62.99, *SD* = 7.53) was significantly different from the group of participants with upper secondary education diploma (*M* = 60.44, *SD* = 7.86) and from the group of participants with higher education diploma (*M* = 57.11, *SD* = 8.18), indicating a significant tendency to score lower on BPO if a higher educational level is achieved. This result may indicate that refining knowledge and developing critical thinking, leads to diverge from methods and values of Black Pedagogy. No significant difference in BPO scores emerged if comparing different kinds of marital relationships in which participants were involved at the moment of administration. For what concerns instead data on nationality, no analyses were conducted to explore differences between Italian and non-Italian participants since the subgroups were too inhomogeneous in number.

Total scores of the double list in ED section concerning diffusion of Black Pedagogy methods in the past (*M* = 32.61, *SD* = 7.13) and nowadays (*M* = 22.53, *SD* = 5.51) differ significantly according to results of paired-samples *t*-test: *t*(802) = 46.36, *p* < .001, and are positively correlated (*r* = .55, *p* < .001).

The item of temporal collocation, modified for the final version of the scale, was positioned as first item of the questionnaire and permitted to assess which generation respondents had in mind when answering to the ED later section (cf. Appendix). The sample of the main study mostly identified the generation of their parents (37.8%) or of their grandparents (36.3%) as the last one in which “old fashioned” educational practices were applied. A small proportion indicated that such methods were used until their great-grandparents generation and no further (4.2%). An unexpected 20.1% declared that respondents’ own generation has witnessed the application of educational methods commonly referred to in our territory as those “old fashioned” and defined in the reference literature as Black Pedagogy practices.

Paired sample *t*-tests were conducted on responses concerning past and present diffusion of each Black Pedagogy method. All the differences between the mean responses resulted significant (*p* < .001), indicating a statistically significant decrease in the diffusion of such practices according to participants’ estimations, consistently with the clear low agreement on “Methods of Black Pedagogy” subscale belonging to prior section. Table 8 summarizes the results of paired samples *t*-test for each comparison.

**Table 8:** Summary of Paired Samples t-Test Results for Each Comparison Between Past and Current Diffusion of Black Pedagogy Methods

Method	Estimated diffusion				Paired differences					*t*	*df*	*p*
	In the past		Nowadays		*M*	*SD*	*SEM*	95% CI of the difference				
	*M*	*SD*	*M*	*SD*				*LL*	*UL*			
Pedagogical beating	3.1	0.77	1.8	0.7	1.3	0.88	.03	1.26	1.38	43.28	825	< .001
Denial of a meal	2.4	0.93	1.2	0.45	1.2	0.92	.03	1.12	1.25	36.91	824	< .001
Cautionary tales	2.9	0.85	1.9	0.73	1.0	0.89	.03	0.94	1.06	32.27	823	< .001
Providing false information	3.1	0.76	2.2	0.77	0.85	0.86	.03	0.79	0.91	27.29	822	< .001
Treating the child coldly	2.9	0.73	2.2	0.78	0.64	0.85	.03	0.58	0.7	21.71	824	< .001
Toughening children up	2.2	0.93	1.2	0.47	0.98	0.9	.03	0.92	1.04	31.37	822	< .001
Monitoring /discouraging sexuality	3.0	0.89	2.0	0.74	1.0	0.91	.03	0.96	1.09	32.32	820	< .001
Lying by exacerbating consequences	2.8	0.8	2.1	0.78	0.71	0.82	.03	0.66	0.77	24.98	818	< .001
Humiliating	2.5	0.89	1.8	0.74	0.77	0.89	.03	0.71	0.83	24.83	824	< .001
Physical violence	2.7	0.9	1.4	0.61	0.85	0.86	.03	0.79	0.9	28.11	823	< .001
Blackmailing	2.6	0.8	2.3	0.83	0.28	0.89	.03	0.22	0.34	8.86	821	< .001
Unpleasant measures for children’s own good	2.9	0.74	2.4	0.8	0.47	0.84	.03	0.41	0.52	15.93	821	< .001

*Note*. 1 = “Not Present at all”; 2 = “Present, but not Common”; 3 = “Present”; 4 = “Widespread”. CI = confidence interval; *LL* = lower limit; *UL* = upper limit.

However, if the specific amounts of such decreases are emphasized, it appears that they are not always similar for all methods. In other words, some clearly decreased more than others. In fact, it can be observed that “Pedagogical beating” is the practice that decreased the most, whereas “Blackmailing” decreased the less. In general, one can see that most methods involving a physical level decreased more than those concerning the psychological area (e.g., treating coldly, humiliating, lying, blackmailing, etc.). Unexpectedly, according to respondents, physical violence decreased less than pedagogical beating, and “Cautionary tales” is one of the methods that decreased more, contrary to what was found in the pilot study where respondents were students with a mean age of 20. More specifically, “Pedagogical Beating” is the method that theoretically could be considered the most representative of the Black Pedagogy construct. On this basis, it is to be presumed that if “Pedagogical Beating” was taken as a reference point, more harmful methods on the physical level (e.g., denial of food, toughening up, and physical violence) should be estimated as less diffused nowadays, whereas psychologically detrimental methods should appear more widespread. As can be seen in Table 9, this supposition is confirmed with the exception of the “Humiliating” method, which seems diffused as much as “Pedagogical Beating”. All the other estimations of diffusion are statistically different from the one regarding “Pedagogical Beating”: according to respondents, physically harmful methods are less diffused (positive mean difference), whereas psychologically detrimental disciplinary practices are more diffused (negative mean difference) than “Pedagogical Beating”.

**Table 9:** Summary of Paired Samples t-Test Results for the Comparisons Between “Pedagogical Beating” Diffusion Nowadays (Maximum Decrease) and Other Methods

Method compared with “Pedagogical Beating”	Paired differences					*t*	*df*	*p*
	*M*	*SD*	*SEM*	95% CI of the difference				
				*LL*	*UL*			
Denial of a meal	0.58	0.7	.02	0.53	0.63	23.68	824	< .001
Cautionary tales	-0.1	0.78	.03	-0.15	-0.05	-3.73	824	< .001
Providing false information	-0.41	0.86	.03	-0.47	-0.35	-13.81	824	< .001
Treating the child coldly	-0.44	0.88	.03	-0.5	-0.38	-14.23	825	< .001
Toughening children up	0.55	0.77	.03	0.5	0.6	20.53	825	< .001
Monitoring/discouraging sexuality	-0.18	0.87	.03	-0.24	-0.12	-5.83	824	< .001
Lying by exacerbating consequences	-0.27	0.86	.03	-0.33	-0.21	-9.03	823	< .001
Humiliating	0.03	0.86	.03	-0.03	0.09	0.9	825	.373
Physical violence	0.36	0.68	.02	0.32	0.41	15.41	825	< .001
Blackmailing	-0.54	0.89	.03	-0.6	-0.48	-17.47	824	< .001
Unpleasant measures for children’s own good	-0.59	0.91	.03	-0.66	-0.53	-18.66	824	< .001

*Note*. CI = confidence interval; *LL* = lower limit; *UL* = upper limit.

Finally, bivariate correlations have been conducted between BPO section total score, its subscales, and the results on the doubled 12-items list of educational practices used in the past and at the present day. Results are summarized in Table 10.

**Table 10:** Summary of Pearson Product-Moment Correlations Between ED Lists, BPO Section and Its Subscales

		BPO subscale		
ED list	BPO total	“Values of Black Pedagogy” (Factor 1)	“Education of children over time” (Factor 2)	“Methods of Black Pedagogy” (Factor 3)
Black Pedagogy in the past	-.076*	-.039	.014	-.153**
Black Pedagogy nowadays	-.130**	-.095**	-.072*	-.140*

*Note*. ED = Estimations of Black Pedagogy Diffusion; BPO = Black Pedagogy Observation.

*p < .05. **p < .01.

As can be seen, correlations do not resemble the results of pilot study, probably because university students do not have children yet. In fact, when parents are responding, a weak negative correlation emerges between the estimation of Black Pedagogy diffusion in the past and the agreement with its methods. It appears that those parents who in the past witnessed a greater diffusion of the educational practices typical of Black Pedagogy, tend to show less agreement with Black Pedagogy methods at the present day and, presumably, with their application to their own children. A result that is in line with the fact, already presented above, that scores on BPO seem to become lower with the increase of age and of the number of children.

## Discussion and Conclusions

The final version of the Black Pedagogy Scale is constituted by a first separated item of temporal collocation, 24 items concerning the detection of Black Pedagogy construct (viz., BPO section) loading on the three factors of “Values of Black Pedagogy”, “Education of children over time” and “Methods of Black Pedagogy”. The second section, namely “Estimations of Black Pedagogy Diffusion” (ED), includes the doubled 12-items list of Black Pedagogy’s practices, which resulted particularly informative although not included in the factorial analysis. The final version of the Black Pedagogy Scale and its factorial structure composed of re-numbered items are provided in the Appendix.

The results of this first examination of the Black Pedagogy Scale seem to be encouraging for a future application of the instrument. The three factors that emerged are consistent with the structure initially hypothesized on the basis of the reference literature. The fact that the mean response on factor “Methods of Black Pedagogy” was the lowest in both studies was an expected result, since policies of children protection have certainly made progresses in Italy after reforms of the Family Law and the ratification of the *Convention on the Rights of the Child*. Nonetheless, the full implementation of the Convention at all levels of our society may not result as a smooth process until individual citizens have completely introjected the image of the child as subject of rights, something that would lead them to have a clear idea of what are the boundaries that have to be respected in child rearing, care and education. Therefore, whereas the most obvious and explicit abusive conducts are correctly identified, adults could still be hindered in recognizing as harmful forms of subtle violence, such as psychological abuse or other kinds of physical disciplinary methods.

Furthermore, even if our results suggest that physically maltreating educational practices of Black Pedagogy are not accepted nowadays, the same cannot be said for the educational values and objectives from which such practices consistently originated. The ongoing diffusion of the subtlest forms of disciplinary methods that are definable as psychologically harmful could be therefore due to the persistence of an obsolete hierarchical and authoritarian conception of the right way to raise and educate children. Nowadays, the threshold of “acceptable” mental or physical violence in an educational relationship seems to be lower than in the past, but the impression is that not all forms of violence are subjected to the same rate of decrease in their use. In fact, responses on the doubled 12-items list regarding estimations of diffusion made by participants, give the impression that psychologically harmful educative and disciplinary practices have not decreased as much as physical ones. In our opinion, this result suggests two possible scenarios: if adults are pursuing educational ideals without being aware that these are consistent with abusive disciplinary methods, they could either apply a level of disciplinary violence deemed acceptable in their cultural and social context (e.g., a slap, a verbal insult, etc.) or, in the best case, they could feel deprived of the means to carry out their educational duty towards children. Both these possibilities indicate that if a change at the level of child-rearing and educational practices is desired, intervention should be placed at the level of values and objectives in order to change them and to allow the spontaneous emergence of different methods. Such consideration could have important implications in interventions aimed at promoting healthy relationships between adult and children. Moreover, the fact that a higher educational level is associated with lower scores on BPO section could help in identifying populations that are most at risk and in designing targeted intervention aimed at interrupting the intergenerational transmission of such practices and values.

The present study shows some limitations that suggest some possible lines of future research. In fact, concurrent validity has still to be assessed, as well as the reliability of the instrument with subjects of different cultures. As mentioned above, the topic concerning the difference in scores on the BPO section between parents raising an only child and parents of more than one child deserves a further in-depth investigation. Moreover, it could be worth exploring the responses of parents with children of different age groups (e.g., preschoolers, adolescents, etc.), and of adults who work in educational contexts with different roles (e.g., teachers, educators, sport trainers, child advocates, etc.) in order to use Black Pedagogy as a further interpretative construct of educational practices, thus designing possible projects of intervention in different contexts.

## Appendix

### The Black Pedagogy Scale

This translation is provided for presentation purposes only: it should not be used for administration because the instrument has yet to be validated in English.

#### Child-Rearing in the Past and Nowadays

In your opinion, which generation was the last to apply “old fashioned” educational practices?

Parents
Grandparents
Great-grandparents
My own generation

Here follow some statements about practices concerning the education of children drawn from the psychological and pedagogical literature on the topic. Read each sentence and then please mark with a cross the number indicating your level of agreement with each statement, according to the following response scale:

**1 = Fully disagree** (You fully disagree with the statement)

**2 = Slightly agree** (You mainly disagree with the statement, but you find yourself in agreement with a part of it)

**3 = Agree** (The statement is close to your thought)

**4 = Fully agree** (The statement reflects exactly what you think)

**Table:**

**1**	Today’s children are more ill-mannered than those of my generation	1	2	3	4
**2**	Children need to learn to unconditionally obey adults who take care of them (parents, teachers, etc.)	1	2	3	4
**3**	The way in which children are educated has nowadays changed for the better	1	2	3	4
**4**	Today’s children show less sense of gratitude towards adults who take care of them (parents, teachers, etc.)	1	2	3	4
**5**	Children’s character should be shaped according to the rules and values of society	1	2	3	4
**6**	Discipline is a fundamental value to be passed down to children	1	2	3	4
**7**	Bad habits and character flaws must be eliminated through education	1	2	3	4
**8**	Today’s children respect the “No” of parents and teachers	1	2	3	4
**9**	Children must respect authoritarian power-holders of a certain context (school, family, etc.)	1	2	3	4
**10**	Pedagogical beatings are sometimes necessary (slapping, caning, etc.)	1	2	3	4
**11**	Children should be kept constantly under control	1	2	3	4
**12**	The value of honesty should be taught to children as early as possible	1	2	3	4
**13**	Children must learn to be humble	1	2	3	4
**14**	Punishment and confiscation are effective disciplinary means	1	2	3	4
**15**	Children must learn to show gratitude and thankfulness for what is being done for them	1	2	3	4
**16**	It is essential to teach children tidiness and cleanliness from a very young age	1	2	3	4
**17**	Children must learn to be diligent and willing to face the tasks they have been entrusted with	1	2	3	4
**18**	Words are always more effective than pedagogical beating	1	2	3	4
**19**	Children’s interest towards the sphere of sexuality should be discouraged	1	2	3	4
**20**	Every error or disobedience must be followed by a corrective measure, or the child will not be coherently brought up	1	2	3	4
**21**	The way in which children are educated has nowadays changed for the worse	1	2	3	4
**22**	It is necessary to show children one’s own inflexibility to be obeyed. Otherwise children will not cooperate	1	2	3	4
**23**	The most effective punishments are those that embarrass children in front of others (classmates, relatives, family members, etc.)	1	2	3	4
**24**	Children must be submissive to parents	1	2	3	4

**Please refer now to the following response scale:**

**1 = Not present at all**

**2 = Present, but not common**

**3 = Present**

**4 = Widespread**

**Table:**

**25**	**In your opinion, how much were the following measures widespread as “old fashioned” educational means?**				
	**a.** Pedagogical beating (slaps, to hit with a stick, etc.)	1	2	3	4
	**b.** Denial of meals or having these replaced with bread and water	1	2	3	4
	**c.** Cautionary tales focused on distressing characters in order to be obeyed (the boogeyman, ghosts, legends, etc.)	1	2	3	4
	**d.** Providing false information to divert from topics mentioned by the child but considered inappropriate for his/her age	1	2	3	4
	**e.** Treating the child coldly as a consequence of his/her disobedience	1	2	3	4
	**f.** Toughening children up in respect to physical exertions, namely to improve their stamina towards fatigue, heat, cold, hunger and tiredness	1	2	3	4
	**g.** Monitoring and discouraging children’s curiosity towards their own sexuality;	1	2	3	4
	**h.** Lying by exacerbating the consequences of a conduct considered wrong with the intention of scaring the child and thus preventing his/her attempts to put such conduct into practice;	1	2	3	4
	**i.** Humiliating: involving other people (family members, relatives, classmates, etc.) in showing disapproval of the child in response to his/her mistake or disobedience;	1	2	3	4
	**l.** Physical violence (beatings, whipping, etc.);	1	2	3	4
	**m.** Blackmailing the child to make him/her do something;	1	2	3	4
	**n.** Always highlighting that when unpleasant measures are executed this is done solely for children’s own good.	1	2	3	4
	**o. Other: _________________________________________________** (You can add here an additional educational method “of the past”)	1	2	3	4

**Table:**

**26**	**According to your experience, to what extent “old fashioned” educational means are still in use nowadays?**				
	**a.** Pedagogical beating (slaps, to hit with a stick, etc.)	1	2	3	4
	**b.** Denial of meals or having these replaced with bread and water	1	2	3	4
	**c.** Cautionary tales focused on distressing characters in order to be obeyed (the boogeyman, ghosts, legends, etc.)	1	2	3	4
	**d.** Providing false information to divert from topics mentioned by the child but considered inappropriate for his/her age	1	2	3	4
	**e.** Treating the child coldly as a consequence of his/her disobedience	1	2	3	4
	**f.** Toughening children up in respect to physical exertions, namely to improve their stamina towards fatigue, heat, cold, hunger and tiredness	1	2	3	4
	**g.** Monitoring and discouraging children’s curiosity towards their own sexuality;	1	2	3	4
	**h.** Lying by exacerbating the consequences of a conduct considered wrong with the intention of scaring the child and thus preventing his/her attempts to put such conduct into practice;	1	2	3	4
	**i.** Humiliating: involving other people (family members, relatives, classmates, etc.) in showing disapproval of the child in response to his/her mistake or disobedience;	1	2	3	4
	**l.** Physical violence (beatings, whipping, etc.);	1	2	3	4
	**m.** Blackmailing the child to make him/her do something;	1	2	3	4
	**n.** Always highlighting that when unpleasant measures are executed this is done solely for children’s own good.	1	2	3	4
	**o. Other: ___________________________________________________** (Write here again the educational method “of the past” that you added in the previous question and now indicate how much is still used nowadays)	1	2	3	4

#### Table of Factors

**Table A1:** BPO Section: Final Factorial Structure With Re-Numbered Items (ML EFA -Varimax Rotation)

Item re-numbered	Values of Black Pedagogy (Factor 1)	Education of children over time (Factor 2)	Methods of Black Pedagogy (Factor 3)
13	.590		
15	.551		
9	.539		
12	.539		
7	.533		
2	.517		
5	.465		
16	.442		
20	.419		
6	.411		
11	.395		
17	.392		
21		.762	
1		.665	
4		.627	
3 (reversed)		.571	
8 (reversed)		.433	
10			.591
22			.506
14			.485
23			.465
18 (reversed)			.447
24			.369
19			.353
